# Effect of a chemical manufacturing plant on community cancer rates

**DOI:** 10.1186/1471-2458-5-34

**Published:** 2005-04-05

**Authors:** Trish Mannes, Katy Emmett, Alan Willmore, Tim Churches, Vicky Sheppeard, Jill Kaldor

**Affiliations:** 1 New South Wales Public Health Officer Training Program, Centre for Epidemiology and Research, NSW Department of Health, LMB 961, North Sydney NSW, 2059 Australia; 2 New South Wales Biostatistical Officer Training Program, Centre for Epidemiology and Research, NSW Department of Health, Sydney, Australia; 3 Centre for Epidemiology and Research, NSW Department of Health, Sydney, Australia; 4 Environmental Health Branch, NSW Department of Health, Sydney, Australia

## Abstract

**Background:**

We conducted a retrospective study to determine if potential past exposure to dioxin had resulted in increased incidence of cancer in people living near a former manufacturing plant in New South Wales, Australia. During operation, from 1928 to 1970, by-products of the manufacturing process, including dioxin and other chemical waste, were dumped into wetlands and mangroves, discharged into a nearby bay and used to reclaim land along the foreshore, leaving a legacy of significant dioxin contamination.

**Methods:**

We selected 20 Census Collector Districts within 1.5 kilometres of the former manufacturing plant as the study area. We obtained data on all cases of cancer and deaths from cancer in New South Wales from 1972 to 2001. We also compared rates for some cancer types that have been associated with dioxin exposure. Based on a person's residential address at time of cancer diagnosis, or at time of death due to cancer, various geo-coding software and processes were used to determine which collector district the case or death should be attributed to. Age and sex specific population data were used to calculate standardised incidence ratios and standardised mortality ratios, to compare the study area to two comparison areas, using indirect standardisation.

**Results:**

During the 30-year study period 1,106 cases of cancer and 524 deaths due to cancer were identified in the study area. This corresponds to an age-sex standardised rate of 3.2 cases per 1,000 person-years exposed and 1.6 deaths per 1,000 person-years exposed. The study area had a lower rate of cancer and deaths from cancer than the comparison areas. The case incidence and mortality due to lung and bronchus carcinomas and haematopoietic cancers did not differ significantly from the comparison areas for the study period. There was no obvious geographical trend in ratios when comparing individual collector districts to New South Wales according to distance from the potential source of dioxin exposure.

**Conclusion:**

This investigation found no evidence that dioxin contamination from this site resulted in increased cancer rates in the potentially exposed population living around the former manufacturing plant.

## Background

The former Union Carbide site at Rhodes was used for manufacturing chemicals from 1928 until 1986, including the timber preservative creosote, xanthates, the pesticide DDT and herbicides (2,4-dichlorophenoxyacetic acid and 2,4,5-trichlorophenoxy acetic acid). Dioxin and other chemical wastes, which were by-products of the manufacturing processes for some of the above, were dumped into the estuarine wetlands and mangroves along the Rhodes peninsula foreshore until 1970 when it was discovered that these by-products were highly toxic. Until this time dioxin effluent was also discharged into Homebush Bay and dioxin contaminated solid waste was used to reclaim land along the Rhodes foreshore.

Dioxin is a generic name that refers to a group of persistent chlorinated contaminants (polychlorinated dibenzo-*p*-dioxins, PCDD's, and polychlorinated dibenzofurans, PCDF's), which are unintended by-products of many industrial activities, such as combustion processes, including power generation, metal works and waste incineration, as well as certain types of chemical manufacture. Dioxin is also produced as a result of some natural processes such as bushfires (forest fires) and volcanic activity. Dioxin is well known for its association with a number of adverse health effects, notably cancer. The most toxic of this group of chemicals is 2,3,7,8-tetrachlorodibenzo-*p*-dioxin (2,3,7,8-TCDD), which has been classified as a Class 1 human carcinogen by the International Agency for Research on Cancer (IARC)[1].

There is concern amongst residents of the Rhodes Peninsula that potential past exposure to dioxin could have resulted in increased incidence of cancer in people living around the Rhodes peninsula. The purpose of this study, therefore, is to determine if potential exposure to dioxins and other pollutants released by the former Union Carbide plant resulted in any difference in the historical incidence of, and mortality due to, cancer in people living on or around the Rhodes peninsula, compared to other people in NSW.

## Methods

### Selection of study area

The suburb of Rhodes is located on a peninsula in the Parramatta River approximately 12 km west of Sydney Harbour and the central business district, in the state of New South Wales (NSW), Australia. Based on the presumed distribution of past dioxin contamination, the study area was defined as the immediate surrounds of the source site as well as areas directly across the river. Australian Bureau of Statistics (ABS)[2] Census Collector Districts were used to define the study area, as these are the finest level of geographical aggregation, containing approximately 200 households, for which age- and sex-specific population estimates are available. Census Collector Districts (CDs) are defined for each quinquennial Census and are current only at Census time[2].

Collector districts were selected for inclusion in the study area if the majority of the geographic extent of the Collector District fell predominantly within a 1.5 km radius from the Union Carbide site. Where inclusion of a Collector District was marginal based on the distance from the site it was included if the outer boundaries were consistent with boundaries for CDs across censuses from 1971 to 2001. Twenty collector districts were chosen from the 2001 Census. The boundaries for the 1976–1996 censuses are consistent, but there are small changes for the 1971 and 2001 Censuses, covering either end of the study period. All selected CDs fell within the Statistical Local Areas (SLA) of Ryde and Concord. SLA is an aggregate of CDs based on local government boundaries.

### Selection of cancer cases

We obtained data on all cases of cancer and deaths from cancer in NSW from 1972 to 2001 from the NSW Central Cancer Registry, which has recorded all cases of malignant neoplasm diagnosed in NSW residents since 1972. Data collected include identifying and demographic information as well as the anatomical and histological characteristics of the disease in each case[3]. Based on a person's residential address at time of cancer diagnosis, or at time of death due to cancer, various geo-coding software and processes were used to ascertain the geographic location of each case or death expressed as latitude and longitude coordinates, using the GDA94 projection. These coordinates were then used to determine to which CD the case or death should be assigned.

### Geographical mapping of cancer cases

The first method used to determine geographic location was MapInfo's GeoLoc product[4]. This is a commercial geo-coding product that attempts to match street addresses with a spatial reference database of streets and approximate street number distributions. Any addresses with postal codes of interest (2112, 2114 and 2138) that were unable to be matched by GeoLoc were then investigated individually using MapInfo's MapMarker software[5], which is a newer geo-coding product of similar design from the same vendor. GeoLoc was used first because some of the authors had found on past projects that the address matching algorithms were superior to those found in MapMarker, although the latter uses a more recent reference database. Addresses that were only approximately matched by either GeoLoc or MapMarker were verified manually. Approximately matched addresses are those in which it was necessary to either ignore one or more of street number, street name, street type or suburb name, or to use phonetic encoding of street details in order to obtain a match against the spatial database.

At the conclusion of this process any addresses still unmatched were then looked up in a file of land parcel information (a cadastral database) supplied by the NSW Department of Lands[6]. Finally, any remaining unmatched addresses were investigated, using a street directory and a motor vehicle, to determine whether they fell within the area of interest. Addresses that were not located (21 addresses) were excluded.

### Population data

A population breakdown by sex and five-year age group was obtained for each quinquennial Census between 1971 and 2001 for all of NSW, Ryde and Concord SLAs and each of the Collector Districts in the study area. Population counts were used to calculate age-sex standardised incidence ratios to compare the study area to the two standard populations: all of the state of NSW (approximately 6 million people) and the statistical local areas (SLAs) of Ryde and Concord that were not part of the study area. Estimated Resident Populations (ERPs) for 30 June each year for NSW and Ryde and Concord SLAs, were obtained from the Australian Bureau of Statistics (ABS).

The Estimated Resident Population (ERP) is an estimate of the Australian population based on results of the quinquennial Census and is compiled as at 30 June of each year. Inter-Censal ERPs are updated using demographic statistics (births, deaths, overseas and interstate migration) and estimates of housing growth or decline[2]. ERPs are not available at the collector district level, thus unadjusted Census counts were obtained for each Census between 1971 and 2001.

An adjustment to the population figures was required for the collector district that includes Concord Repatriation General Hospital (2001 Collector District 1410103). The NSW Cancer Registry, which provided the numerator data for this study, records residential addresses at time of cancer diagnosis, or death. On the other hand the Census, which provided the denominator data for this study, records all people present on Census night at a particular location, including people in Concord Hospital. We thus attempted to estimate the population of the collector district minus the hospital population. We obtained the average number of occupied beds for each year from hospital and health service annual reports. The age-sex structure of this population was then estimated using data from the NSW Inpatient Statistics Collection[7], which is a complete enumeration of hospital admissions in NSW. Data for Concord hospital is only available for 1993 onwards; the year it became integrated into the State Health System, thus 1993 proportions were used for each census between 1971 and 1991.

### Data analysis

Indirect standardisation was used to create various age-sex standardised incidence and mortality ratios (SIRs and SMRs)[8]. Ninety-five per cent confidence limits were constructed around these ratios assuming a Poisson distribution of errors[9]. SIRs and SMRs were calculated for all cancers and for lung and bronchus carcinoma and haematopoietic cancers (such as lymphomas and leukaemias). Dioxin is known to act primarily as a promoter of all neoplasms, based on observations in cell and animal models[1]. However, end-points of carcinoma of the lung and bronchus, and haematopoietic neoplasms were chosen for closer investigation as evidence for increased risk of these cancers were found in occupational cohort studies used by the IARC in the evaluation of the carcinogenicity of 2,3,7,8-TCDD[1]. Cases of carcinoma of the lung and bronchus and haematopoetic cancers were identified using the International Classification of Diseases – Tenth revision (ICD-10) codes.

The area that the 30-year SIRs and SMRs were based on was not completely consistent over the whole study period as the collector district boundaries changed over time. Changes to collector district geographic boundaries occurred between the 1971 and 1976 Censuses and between the 1996 and 2001 Censuses. Thus SMRs and SIRs based on these boundaries included a number of cases (or deaths) that were not enumerated on the same basis as the population for all time periods. To check that these discrepancies did not bias the results, a 25-year count covering the period 1974–1998 was also calculated for comparison. This used 1996 boundaries, which were consistent for each census going back to 1976.

All analysis and data manipulation was carried out using the SAS System^® ^version 8.02 and Microsoft^® ^Excel 2000.

## Results

The study area surrounding the Union Carbide site at Rhodes contains 20 Census Collector Districts, representing a resident population of 11,800 people as at the 2001 Census. The population fluctuated between approximately 10,800 and 11,800 during the 30 year study period. The median age in the study area was 37 years, whereas the median age in Concord SLA was 36, Ryde SLA was 34 and the median age in NSW was 35 years. 1,106 cases of cancer and 524 deaths due to cancer were identified in the study area during the study period, corresponding to an age-sex standardised rate of 3.2 cases per 1,000 person-years exposed and 1.6 deaths per 1,000 person-years exposed. Table 1 provides details of the most common types of cancers observed in the study area during the study period.

**Table 1:** Number (and percentage) of cases of selected types of cancers occurring in the Rhodes study area compared with New South Wales (based on 30 year counts).

***Cancer type***	***Rhodes (number of new cases)***	Percentage
Lung and Bronchus Carcinomas	143	12.93%
Haematopoietic	85	7.69%
Leukaemia	31	
Non-Hodgkin's Lymphoma	40	
Multiple Myeloma	9	
Prostate	116	10.49%
Breast cancer	142	12.84%
Colorectal	153	13.83%
Gynaecological	61	5.52%
Urothelial	35	3.16%
Melanoma of the skin	94	8.50%
Site unknown	57	5.15%

Definitions:

Colorectal: Cancers of colon, rectum and anus (ICD-10 C18-C21)

Haematopoietic: Lymphoma, leukaemia, multiple myeloma (ICD-10 C81-C96)

Gynaecological: Uterus, ovary, other female genital organs (ICD-10 C51-C58)

Urothelial: Urinary tract excluding body of kidney (ICD-10 C65-C68)

Table 2 presents the results of SIR and SMR analyses for 30 year and 25 year group counts for all cancers. Aggregated over the entire study period, the study area had a significantly lower rate of cancer than the whole of NSW (SIR = 88%, 95%CI 83–93). The study area also had a significantly lower rate of death due to cancer than the whole of NSW during the entire study period (SMR = 89%, 95%CI 81–97).

**Table 2:** SIR and SMR for Rhodes study area compared with New South Wales and compared with Ryde and Concord SLAs for all cancers.

***Time Frame***	***Number of cases/ deaths***	***Comparison Area***	***Case SIR (Indirectly Standardised)***	***95% Confidence Interval***	***Death SMR (Indirectly Standardised)***	***95% Confidence Interval***
30 years	1106/524	New South Wales	87. 6#	(82.5, 92. 9)	88.7#	(81.3, 96.7)
30 years	1106/524	Concord and Ryde SLAs	90.5#	(85.2, 96.0)	92.9	(85.1, 101.2)
25 years	922/530	New South Wales	91.2#	(85.4, 97.2)	93.5	(85.2, 102.3)

Notes:

SLA = Statistical Local Area

SIR = Standardised Incidence Ratio

SMR = Standardised Mortality Ratio

# Statistically significantly lower than the comparison area

* Statistically significantly higher than the comparison area

A comparison was also made to the numbers expected if the rate observed in the balance of the SLAs of Ryde and Concord was experienced in the study area. The case incidence in the study area was statistically significantly lower than the rate in the surrounding local area (SIR = 91%, 95%CI 85–96), while the death rate was marginally lower (SMR = 93%, 95%CI 85–101).

The 25 year results (Table 2) were similar to the results obtained using 30 year counts, suggesting that the slight variation in the definition of the study area used for the 30 year period did not bias the results.

The yearly SIRs for the study area compared to NSW varied between 51% (95%CI 50.0–80.0) in 1976 and 130% (95%CI 97.2–107.6) in 1984, while the yearly SMRs were in the range 43% (95%CI 19.8–82.3) in 1991 to 124% (95%CI 82.0–181.0) in 1984. There was no obvious trend in ratios with apparently random, symmetrical fluctuation around the overall 30-yr averages of 87.6% (95%CI 82.5–92.9) and 88.7% (95%CI 81.3–96.7), for SIR and SMR respectively. The count of cases and deaths that determined these ratios varied between 20–50 cases and 10–30 deaths per year.

Similarly, when groups of years were aggregated around census years for year-block analysis there was no obvious pattern in the SIR and SMR for the study area compared to NSW. The SIR ranged from 77% (95%CI 64.5–91.2) in the period 1974–1978 to 99% (95%CI 85.3–113.0) in the period 1984–1988, while the SMR varied between 76% (95%CI 60.4–94.7) in the period 1989–1993 to 108% (95%CI 89.0–129.4) in the period 1984–1988.

Table 3 shows SIR and SMR for the individual CDs compared to NSW. The SIR for the individual CDs compared to NSW varied between 49% (95%CI 25–84) and 184% (128–256), while the SMR ranged from 22% (95%CI 3–78) to 137% (95%CI 102–181). Again, there was no obvious trend in ratios with symmetrical fluctuation around the overall total study area 25 year averages of 91% (95%CI 85–97) and 94% (95%CI 85–102), for SIR and SMR respectively. There was no apparent relationship between SIR, SMR and distance of the CD relative to the source of dioxin contamination.

**Table 3:** SIR and SMR for each census collector district in the Rhodes study area compared with New South Wales for all cancers (based on 25 year counts).

***Geographical Region***	***Number of cases***	***Distance from site*^+ ^*(metres)***	***Case SIR (Indirectly Standardised)***	***95% Confidence Interval***	***Death SMR (Indirectly Standardised)***	***95% Confidence Interval***
1410101	76	200	113.6	90.3–141.0	119.0	85.4–161.4
1410102	34	985	149.0*	108.7–199.3	113.8	66.3–182.3
1380508	14	1014	49.0#	26.8–82.3	52.3	19.2–113.9
1380507	13	1190	48.5#	25.0–84.6	21.7#	2.6–78.2
1380503	35	1241	96.3	67.8–132.7	111.2	68.0–171.8
1380509	58	1248	184.1*	128.3–256.1	128.2	61.5–235.8
1380501	76	1250	76.3#	57.8–98.9	66.8#	42.8–99.3
1410103	78	1302	91.3	74.0–111.5	81.9	58.8–111.1
1381207	61	1415	80.1	61.1–103.1	79.5	54.0–112.9
1381209	76	1428	102.8	81.9–127.4	137.4*	102.3–180.6
1380505	25	1527	94.3	63.2–135.5	107.7	58.9–180.6
1380504	58	1530	83.0	63.5–106.6	93.1	66.2–127.2
1380510	51	1561	71.8#	51.3–97.8	79.9	52.2–117.1
1381211	38	1576	101.3	72.4–138.0	83.5	46.7–137.7
1380506	22	1633	100.9	63.2–152.8	116.7	58.3–208.8
1381210	11	1638	56.3	28.1–100.6	88.3	38.1–174.0
1410104	8	1682	84.5	56.2–122.2	93.4	52.3–154.1
1380502	34	1717	105.8	80.2–137.1	109.1	72.5–157.6
1410105	71	1874	89.6	68.8–114.6	99.0	68.6–138.3
1410301	83	1935	81.1	63.6–102.0	82.6	58.2–113.9

Notes:

SLA = Statistical Local Area

SIR = Standardised Incidence Ratio

SMR = Standardised Mortality Ratio

+ Distance from site = distance between the centroid of the collector district from the centre of the former Union Carbide site

# Statistically significantly lower than the comparison area

* Statistically significantly higher than the comparison area

Table 4 shows the SIR and SMR for carcinoma of the lung and bronchus and haematopoietic carcinoma. Over the 30 year study period there were 143 cases and 121 deaths due to carcinomas of the lung and bronchus identified in the Rhodes study area. The case incidence and mortality ratios due to lung and bronchus carcinoma were not significantly different to NSW or to the statistical local areas of Ryde and Concord for the study period.

**Table 4:** SIR and SMR for Rhodes study area compared with New South Wales and compared with Ryde and Concord SLAs for lung and bronchus carcinoma and haematopoietic cancers (based on 30 year counts)

***Cancer type***	***Number of cases/ number of deaths in Rhodes study area***	***Comparison Area***	***Case SIR (Indirectly Standardised)***	***95% Confidence Interval***	***Death SMR (Indirectly Standardised)***	***95% Confidence Interval***
Lung and Bronchus Carcinomas	143/121	NSW	105.5	88.9–124.2	107.1	88.9–128.0
Haematopoietic neoplasms	85/46	NSW	80.6#	64.3–99.6	80.8	59.2–107.8
Lung and Bronchus Carcinomas	143/121	Concord & Ryde SLAs	115.7	97.5–136.3	116.2	96.4–138.8
Haematopoietic neoplasms	85/46	Concord & Ryde SLAs	75.0#	59.9–92.7	78.3	57.3–104.4

Notes:

SLA = Statistical Local Area

SIR = Standardised Incidence Ratio

SMR = Standardised Mortality Ratio

# Statistically significantly lower than the comparison area

* Statistically significantly higher than the comparison area

Over the 30 year study period there were 85 cases and 46 deaths due to the haematopoietic neoplasm identified in the Rhodes study area. The case incidence of the haematopoietic class of cancers in the Rhodes study area was marginally statistically significantly lower than NSW and Ryde and Concord over the study period. There is no significant difference in the mortality due to this class of cancers.

## Discussion

We examined age-sex standardised incidence and mortality ratios due to cancer in the 20 Collector Districts within 1.5 km from the former Union Carbide site (the study area). We used incidence and mortality for all of NSW and for the balance of the Ryde and Concord Statistical Local Areas, which enclose the study area, as the comparison. We calculated standardised incidence and mortality ratios for all cancer as well as carcinoma of the lung and bronchus and haematopoietic neoplasm. Annual, twenty-five year and thirty year comparison periods were used. We found that the incidence and mortality ratios for all types of cancer for all comparison periods of study in the study area did not differ significantly from expectation, based on cancer incidence and mortality rates in all of NSW and in the areas immediately surrounding the study area.

There are a number of strengths to this study. We were able to obtain information on all cases of and deaths from cancer in the study and control areas during the study period as the NSW Central Cancer Registry is supported by compulsory notification of all cancers in NSW. We selected those collector districts whose population fell within 1.5 km of the former Union Carbide site. The study area included 20 Collector Districts with a population of approximately 11,500 people. Since there was data available for a number of years we were able to examine cancer incidence and mortality in individual Collector Districts, demonstrating that even the residential areas closest to the site did not have increased cancer rates.

The study did present several challenges, however. As with any small area study, one of the biggest difficulties was the definition of study boundaries, which are consistent over a sufficiently long period to accumulate a reasonable number of cases or deaths. This was a challenge for a number of reasons. Firstly, the exposed community was not easily defined, because the potential exposure route was unclear. Historically there was no known exposure via air. Potential exposure routes may have been contact with Homebush Bay, eating fish caught in the bay, soil transferred from the site or occupational exposure from working at the facility. It is also possible that no exposure to dioxins occurred in residents surrounding the Union Carbide plant.

Secondly, the choice of possible boundaries was limited to those for which denominator (population) counts were available, as these were needed to calculate expected counts of cases and deaths due to cancer. The smallest units for which population data are available are the ABS Census Collector Districts. Unfortunately, these boundaries were still too coarse to enable definition of a study area with a circumference that was a uniform distance from the putative exposure source. Also, the ABS may change the boundaries of Collector Districts at each Census in response to significant changes in population. Fortunately for 25 of the 30 years of this study the boundaries remained consistent allowing direct comparisons across time periods.

The geo-coding process used to determine if a case belonged to the exposed study area presented some difficulties. Although the address information for each case was quite "clean" and well formatted, only eighty one percent (4592 out of 5644) of the cases falling into the postcodes containing the study area could be matched exactly using an automatic process. On further investigation, approximately seven percent of cases originally attributed to the study area, but not matched exactly, were re-allocated to another Collector District. However, overall the total number of cases falling into the study area changed by only 2 (out of 1106). The number of cases where the address was unable to be found, and therefore the case excluded from the study area, was a very small proportion of the total number of cases recorded in these postcodes (21 cases out of 5644, or 0.4 percent).

An additional challenge in this study was assigning population estimates to areas for the purpose of calculating expected numbers of cases and deaths. The methods used to assign population estimates varied between the study and control areas. Since yearly population estimates (in the form of ERPs) were not available at the Census Collector District level, Census year enumerated population counts were used and extrapolated to inter-Censal years. ERPs (from the Australian Bureau of Statistics) were used for NSW and the SLAs of Ryde and Concord and more closely reflect actual population. The use of enumerated usual resident populations in the study area instead of ERPs may result in small errors in the SIRs and SMRs. However, the magnitude of these errors is unlikely to be large enough to affect the results of the study. Additionally, population figures for the Collector Districts, which included Concord Hospital, overestimate the residential population due to counting of hospital inpatients on Census night. The population figures were adjusted by subtracting the estimated number of people at Concord Hospital on Census night, thus providing a better estimated residential population in this Collector District.

Another limitation of this study is our inability to adjust for potential confounders in the relationship between cancer incidence and mortality and place of residence. Potential confounders include smoking rates and socio-demographic variables. The comparison of the study area to the balance of the SLAs of Ryde and Concord may compensate in part for this deficiency, as this area, which is contiguous with the study area, is likely to have demographic characteristics which are very similar to the study area.

One of the major weaknesses in small area studies is that the numbers of events being examined are often very small and therefore variation in these numbers can more often be attributed to chance than any exposure effect. This was particularly the case when we looked at annual incidence and mortality. Due to the small numbers of events each year the confidence intervals around the ratios are wide and consequently there are only three significant results, with the SIR for 1974 and 1976, and the SMR for 1991 being significantly lower than 100%. When testing the significance of this many ratios (sixty in total) at the 95 percent confidence level, three significant results are expected purely as a result of chance. Given the negative result no attempt was made to adjust for the effect of multiple comparisons. Based on this and the fact that there is no obvious trend in the yearly SIRs and SMRs we conclude that there is no time effect on the incidence of and mortality due to cancer in the Rhodes area compared to NSW.

It is possible that many workers at the Union Carbide plant resided in the study area. Had occupational exposure to dioxin on the site been high, workers may have experienced greater rates of cancer than the general population and may have raised the observed risk of cancer in our study. The cancer registry does not collect occupational data, thus it was not possible to adjust for such potential confounding.

There are a number of additional issues to be considered when interpreting studies of cancer incidence around a point source of carcinogenic exposure. Cancer risk is likely to be significantly greater for those working at the plant than those residing near to the plant, because potential exposure to carcinogens is likely to be much greater in workers. Accordingly, in residents the same cancers would be expected as those observed in workers, but at lower incidence. Thus, many studies of cancers in residents are negative and may be underrepresented in the literature. In some cases where an increased risk of cancer has been demonstrated in residents, a causal association has not been assumed, as the cancers observed are different to those seen in occupational studies[10], or there is an absence of measured exposure to the contaminant of concern[11].

Human exposure to TCDD, studied in the context of industrial accidents and occupational exposure, is associated with increased risk of cancer (all sites), and in some groups, increased risk of lung cancer, soft tissue sarcoma and non-Hodgkin lymphoma have been observed[1,12]. TCDD, therefore, is considered to be an overall promoter of cancer. Cancer excesses have not been observed in TCDD-exposed cohorts with lower exposures[12]. Increased risk of cancer reasonably attributed to site contamination by TCDD (as distinct from exposure to TCDD consequent upon an industrial disaster or in workers) has never been demonstrated[1]. In the present study we found no evidence that dioxin from this site has resulted in increased cancer rates in the potentially exposed population on the Rhodes peninsula and in the surrounding area. The findings of this study are consistent with the understanding that the risk of cancer attributable to residing near a site contaminated by TCDD is low.

## Conclusion

We did not find a significant increase in the historical incidence of, or mortality due to, cancer for people living around the Rhodes peninsula compared to other people in NSW. The findings of this study are consistent with the understanding that the risk of cancer attributable to residing near a site contaminated by TCDD is low.

## Competing interests

The author(s) declare that they have no competing interests.

## Authors' contributions

TM managed the project in the later stages and prepared the manuscript. KE managed the project in the early stages, including compiling data, geo-coding cases and deaths and calculating summary statistics. KE also prepared an early draft of the manuscript. AW assisted with the geo-coding of cases and deaths using Geoloc and MapMarker software, and the LPI database. TC and VS were involved in planning and advising on the project throughout. JK provided statistical advice throughout the project. All authors provided comment on early drafts of the manuscript and all authors read and approved the final manuscript.

**Figure 1:**
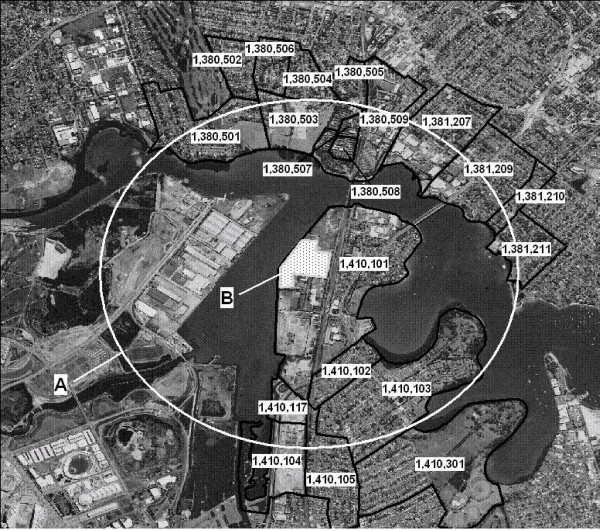
**Map of study area with Census collector district boundaries**. A: Approximate 1.5 km distance from the former Union Carbide plant. B: The site of the former Union Carbide plant. Study Area: Includes all Collector Districts marked on the aerial photograph. Ryde Statistical Local Area: Is located to the north of the study area. Concord Statistical Local Area: Is located to the south of the study area. The area to the west of the former Union Carbide plant is former industrial land. No residential occupation occurred here until approximately the year 2000.

## Pre-publication history

The pre-publication history for this paper can be accessed here:


